# Prospective study of the Transurethral Suprapubic endo-Cystostomy (T-SPEC^®^): an ‘inside-out’ approach to suprapubic catheter insertion

**DOI:** 10.1007/s11255-014-0884-x

**Published:** 2014-11-26

**Authors:** Brian J. Flynn, Robert J. Larke, Paul B. Knoll, Kirk M. Anderson, Vassilis J. Siomos, Andrew P. Windsperger

**Affiliations:** Division of Urology, University of Colorado Denver, Academic Office One Bldg, 12631 East 17th Ave., Room L15-5602, Box C319, Aurora, CO 80045 USA

**Keywords:** Medical device, Urinary bladder, Catheter, Instrumentation, Incontinence

## Abstract

**Objectives:**

To prospectively evaluate the new medical device Transurethral Suprapubic endo-Cystostomy (T-SPeC^®^), used for suprapubic catheter (SPC) placement via the transurethral (inside-to-out) approach, and examine the 30-day outcomes in the first US series.

**Methods:**

IRB approval was obtained for this prospective study. We evaluated the first 114 consecutive cases of SPC placement using the T-SPeC^®^ device by a single surgeon at in a 20-month period. We excluded patients who underwent alternative approaches to suprapubic catheter placement including open abdominal approach (12) and percutaneous approach (5). Preoperative patient demographics, operative detail, success rate and 30-day complication rate were recorded.

**Results:**

We successfully placed an 18 Fr suprapubic catheter using the T-SPeC^®^ device in 98.2 % of patients. During the procedure, the capture housing was missed twice. The mean patient age was 56.6, BMI 29.4 kg/m^2^, skin to bladder distance 6.7 cm and operative time 3.6 min. There were 12 postoperative complications within 30 days of the procedure including urinary tract infections (6), SPC exit site infection (2), SPC blockage (2) and catheter expulsion (2). There were no Clavien–Dindo grade III–IV complications such as re-operation, small bowel injury, hemorrhage or death.

**Conclusion:**

The T-SPeC^®^ device is a novel, simple, accurate and minimally invasive device for SPC insertion from an inside-to-out approach. Our prospective study demonstrates that the T-SPeC^®^ device can be placed safely and efficiently in a variety of patients with a need for urinary drainage.

## Introduction

**Fig. 1:**
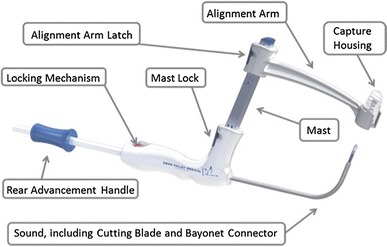
Schematic of the T-SPeC^®^ Surgical System. The rear advancement handle advances the cutting blade (15 Fr) from inside the bladder, through the bladder wall and abdomen, and pulls the catheter connected to the bayonet connector on the coaxial coil back through the surgical pathway for placement in the bladder. The locking mechanism in the rear handle prevents the cutting blade and coaxial coil from being inadvertently advanced. The mast guides the alignment arm to the patient’s abdomen before creation of the surgical pathway. Abdominal thickness can be measured using the graduated mast. The alignment arm holding the capture housing provides the surgeon with the blade exit point. The cutting blade makes a small incision (15 Fr) through the bladder, fascia and abdominal wall. It is housed within the sound and is deployed by the rear advancement handle. The capture housing accepts the surgical blade once it has passed through the patient’s bladder wall and abdomen. The blade and capture housing can be removed for disposal by rotating the capture housing

The Transurethral Suprapubic endo-Cystostomy Device (T-SPeC^®^) is a novel disposable medical device used for insertion of a suprapubic catheter (SPC) via a transurethral approach (Fig. 1). The T-SPeC^®^ device (Swan Valley Medical Inc. Bigfork, MT) has FDA 510(k) clearances. T-SPeC^®^ device compensates for abdominal girth to allow accurate and efficient catheter placement from an ‘inside-out’ direction. T-SPeC^®^ Surgical System comes in two models, T7 and T14. The T7 model can accommodate a bladder to skin distance of up to 7 cm, while the T14 can accommodate 14 cm. T-SPeC^®^ is similar to the Lowsley retractor that was used commonly for suprapubic cystostomy catheterization before the development of percutaneous SPC kits [1]. However, unlike the Lowsley device that requires an incision to cut down to expose the tip of the curved metal sound, the T-SPeC^®^ instrument initiates a 15 Fr surgical tract from within the bladder and exits the skin just cephalad to the pubic bone.

In 2013, the senior author (BJF) and colleagues published a pilot study that demonstrated the proof of concept on a cadaver model as well as initial experience in four men at St. Mary’s General Hospital in Kitchener Ontario, Canada [2]. These live cases were performed in the outpatient setting successfully using the T-SPeC^®^ T7 Surgical System in a mean of 9.7 min under general anesthesia without any complications. Once feasibility was demonstrated and FDA clearance occurred, one of the authors (BJF) elected to study this device in his practice. We prospectively evaluated the new medical device T-SPeC^®^ and examined the 30-day outcomes in the first US series.

## Materials and methods

### Patients

This was an IRB-approved prospective case series (COMIRB #14-0378) of 114 consecutive patients who underwent suprapubic catheter placement with the T-SPeC^®^ device as part of complex genitourinary reconstruction or an isolated procedure. After informed consent, the patient underwent suprapubic catheter placement via the T-SPeC^®^ device by a single surgeon at the University of Colorado Hospital in Aurora, CO, between November 2012 and July 2014. Data were collected prospectively from the time of the preoperative visit until the 30-day postoperative visit.

### Surgical technique

**Fig. 2:**
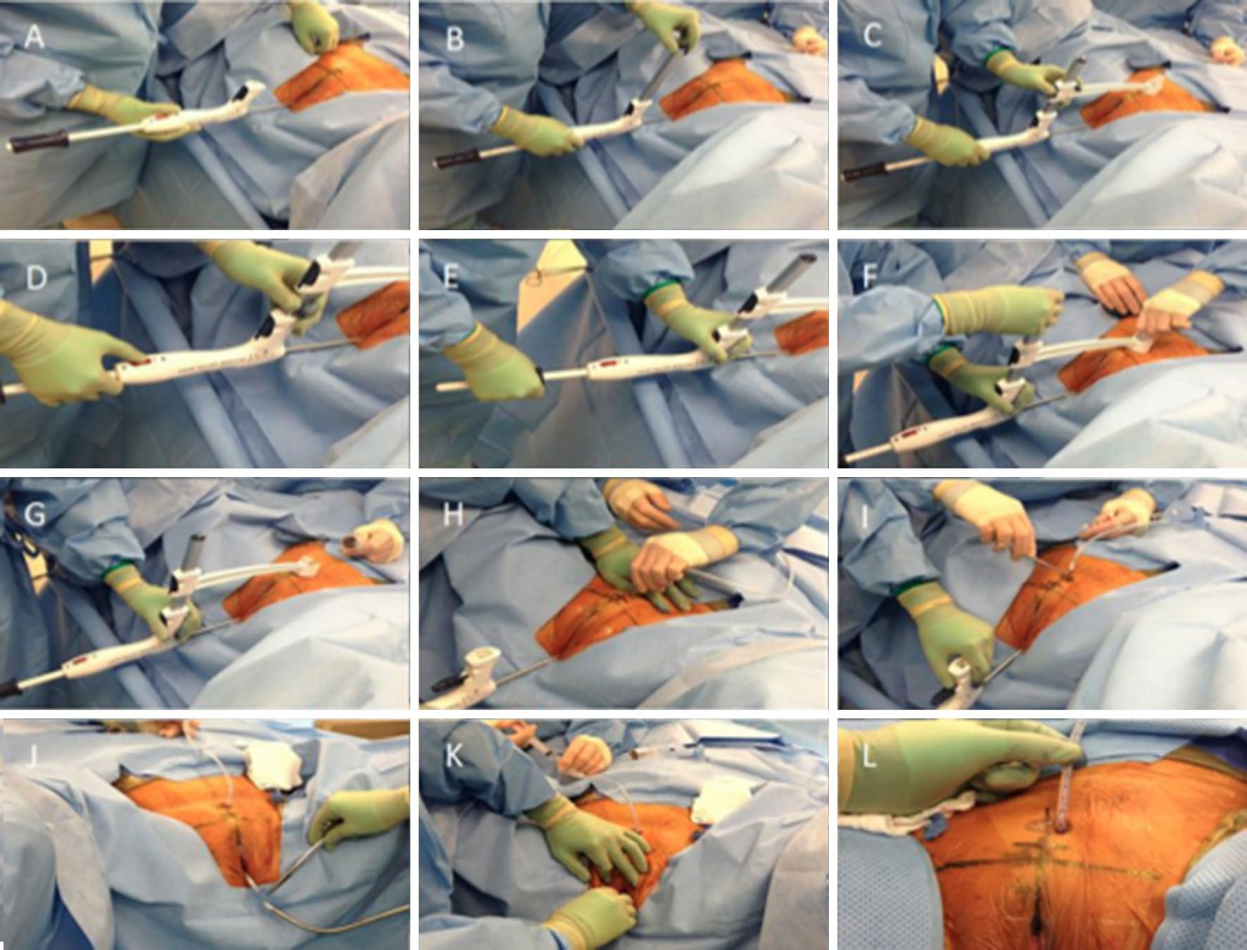
**a–l** Insertion of the sound per urethra and angling of the sound toward the abdominal wall (**a**). Attachment of the mast to the handle (**b**). Positioning of the alignment arm and blade capture housing (**c**). Unlocking the safety to allow advancement of the coaxial coil (**d**). Advancement of the blade attached to a coaxial coil from the tip of the sound through the abdominal wall (**e**). Removal of the capture housing which now contains the perforating blade that cuts through the abdominal wall (**f**–**g**). Attachment of the 18 Fr silicone catheter to the bayonet connector on the coaxial coil above the skin surface (**h**). Spreading of the 15 Fr tract to allow the 18 Fr catheter to pull into the bladder (**i**). Disconnection of the catheter by dividing the catheter where it was affixed to the bayonet connector (**j**). Advancing the catheter tip back into the bladder with a hemostat and inflation of the 10-ml balloon with saline (**k**). Final position of the 18 Fr catheter (**l**)

The procedure commenced after an appropriate anesthetic and antibiotic had been administered. All patients were placed in a dorsal lithotomy position prior to placement of the sound. In the male patient, cystoscopy was performed next to evaluate for urethral pathology. Cystoscopy was not routinely performed prior to placement of the sound on female patients. Figure 2 overviews the key steps of the procedure, the skin is marked 2 cm cephalad to the pubic bone in the midline, mast and alignment arm are attached to the device, which automatically places the capture housing directly over the tip of the internal end of the sound, on the abdominal surface. The internal tip of the sound is palpated under the site mark. The shaft of the T-SPeC^®^ device is then tilted 10° below horizontal, and the safety switch is disabled.

**Fig. 3:**
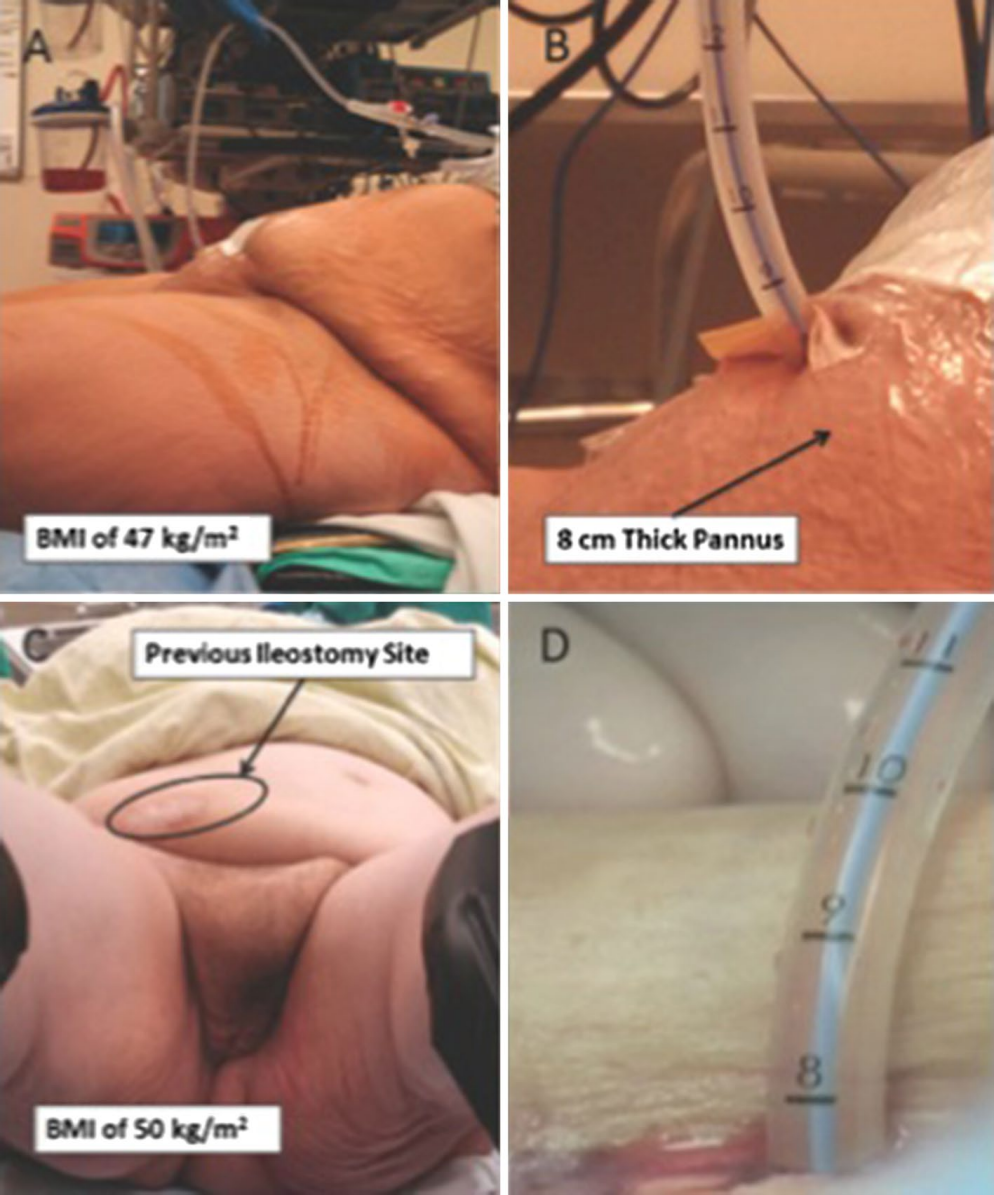
**a** Photograph of a morbidly obese woman (BMI of 47 kg/m^2^) with a neurogenic bladder secondary to multiple sclerosis (**a**). She is in the lithotomy position for suprapubic catheter placement utilizing the T-SPeC^®^ device under local anesthesia. The indications for the procedure were urinary retention and her inability to perform self-catheterization. The T-SPeC^®^ T14 model is recommended in patients with BMI > 35 kg/m^2^ **. b** T-SPeC^®^ device measuring distance from edge of skin to bladder at 8 cm, implying an 8-cm-thick pannus**. c** Photograph of a morbidly obese woman (BMI of 50.2 kg/m^2^) with a neurogenic bladder secondary to CVA. The indications for the procedure were urinary retention and incontinence. Other comorbid conditions included a history of ileostomy and subsequent takedown**. d** T-SPeC^®^ T14 device measuring 8 cm from bladder to skin which exceeds the length of most percutaneous SPC trocars

The advancement handle is moved forward to the designated depth, which sends the cutting blade sequentially through the end of the sound, the bladder, the subcutaneous tissue, and into the capture housing. The blade, alignment arm and mast are then removed. Next, the prepackaged 18 Fr catheter is attached to the bayonet connector. A 1-mm incision is made in the skin adjacent to the sound exiting the skin. The catheter and sound are then pulled into the bladder and then exit at the urethral meatus. The catheter is disconnected and then advanced back into the bladder with a cystoscope. The balloon is inflated with 10 ml of sterile water. Accurate catheter placement is then confirmed by cystoscopy. There is no need to suture the catheter to the skin at the end of the procedure. The distance from the bladder to the skin was measured both intra-operatively and postoperatively by the 1-cm incremental markings on the catheter from the balloon to the hub (Fig. 3). Operative time was recorded from the time of placing the T-SPeC sound in the urethra to the end of the cystoscopy.

## Results

Table 1 details the patient demographics in 114 patients. The indication for SPC placement was chronic bladder drainage in 12 % and temporary postoperative urinary drainage in 88 %. Almost half of the patients (45.6 %) in the study were obese (BMI > 30 kg/m^2^), 12 % of patients had neurologic disease, 15.8 % of patients had a history of recurrent UTIs, and many patients had prior abdominal surgery (appendectomy, ileostomy/colostomy, colectomy, cholecystectomy, gastric bypass or gastric fundoplication). Despite a significant number of patients with neurologic disease and morbid obesity, all patients were placed in lithotomy position. <10 % of patients had preoperative imaging. Those with preoperative imaging included patients undergoing major urinary tract reconstructive procedures such as vesicovaginal fistula, enterovesical fistula, posterior urethroplasty and bladder diverticulectomy.

**Table 1:** Patient demographics

Total number of patients	114
Mean age [years (range)]	57 (33–90)
BMI [kg/m^2^ (range)]	29 (17–50)
Sex	
Male [*N* (%)]	15 (13.2)
Female [*N* (%)]	99 (86.8)
Indications	
Neurogenic bladder [*N* (%)]	14 (12.3)
Incontinence	4
Retention	10
Female reconstructive surgery [*N* (%)]	89 (78.1)
SUI	64
LUT mesh perforation	13
Fistula repair	9
Urethral diverticulum repair	1
Female urethroplasty	2
Male reconstructive surgery [*N* (%)]	11 (9.6)
Bladder diverticulum repair	1
Urethral stricture or fistula repair	10
Comorbid conditions	
Obesity (BMI > 30 kg/m^2^) [*N* (%)]	52 (45.6)
Prior radiation [*N* (%)]	3 (2.6)
Neurologic conditions [*N* (%)]	14 (12.3)
Chronic UTIs [*N* (%)]	18 (15.8)
Previous abdominal surgery	181
Gastrointestinal [*N* (%)]	47 (41.2)
Urological [*N* (%)]	61 (53.5)
Gynecological [*N* (%)]	82 (71.9)

Outcome measures of the T-SPeC^®^ procedure are as follows. The majority of the T-SPeC^®^ procedures used the T7 device (67.5 %) and were performed under general anesthesia (93 %) as most of cases were done in combination with major pelvic surgery. The mean operative time was 3.6 min (range 2.5–5.5 min), and the average abdominal wall thickness was 6.7 cm (4–11 cm). There were only two procedural difficulties (1.8 %). In these two cases, the capture housing was missed. The knife blade was removed with a hemostat, and the catheter was attached to the connector allowing successful placement. A second kit was not required in either case.

The 30-day T-SPeC^®^ were our primary endpoints. The T-SPeC^®^ device was successfully placed and provided bladder drainage for the desired duration in 98.2 % of patients. There were two premature catheter expulsions, and these were considered failures (1.8 %). In both men, the incident was due to patient factors, mental illness in one patient that lead to him pulling the catheter out with the balloon inflated and one patient who had the catheter pulled out while transferring from wheelchair to bed. Neither incident was due to catheter design or malfunction. The majority of patients (89.5 %) did not have any complications in the 30 days following surgery. The most common postoperative complication was a urinary tract infection (UTI), occurring in six patients(5.3 %). Two of the six patients had a history of chronic UTIs. This number compares favorably with the 15.8 % incidence of preoperative recurrent UTIs in our study population. There were two minor wound infections successfully treated with antibiotics. In two patients, the SPC became permanently obstructed postoperatively with urinary sediment thereby requiring a urethral catheter. In these two cases, the initial SPC was left in place for 30 days to establish the tract and then was successfully exchanged for a 20 Fr suprapubic catheter. Most importantly, there were no major complications (Clavien–Dindo grade III–IV) such as bowel injury, vascular injury, death or need for re-operation.

## Comments

Suprapubic cystostomy is commonly used for long-term as well as temporary urinary drainage. Patients requiring long-term urinary drainage would include those with a neurogenic or end-stage bladder who are unable or unwilling to perform clean intermittent catheterization [3]. Patients needing long-term bladder drainage prefer chronic SPC to a Foley as it avoids the risk of urethral erosion [4]. Temporary SPC drainage is preferred by many surgeons for postoperative urinary drainage due to improved patient comfort and ease of performing a voiding trial. For instance, Krane et al. [5] compared suprapubic bladder drainage to Foley drainage in 200 men undergoing robotic-assisted laparoscopic radical prostatectomy (RALP). They noted the patients who had a SPC had significantly decreased catheter-related discomfort and a straightforward voiding trial allowing early catheter removal.

Although urologists recognize the short- and long-term value of SPC, it remains an underutilized procedure due to fear of life-threatening complications such as a bowel injury as well as a lack of a universal method for safe, efficient and accurate placement especially in obese patients [6–9]. Ahluwalia et al. [4] found a 10 % intra-operative complication rate, 2.5 % risk of bowel injury, 19 % 30-day complications rate and a 1.8 % mortality rate in 219 patients who underwent percutaneous suprapubic insertion under cystoscopic guidance. Similarly, the US Department of Health and Human Services, Agency for Healthcare Research and Quality (AHRQ) reported a ten-year average US mortality rate for SPC placement via the ‘outside-in’ methods of 4.4 %. More recently, the British Association of Urologic Surgeons (BAUS) guidelines now recommend fluoroscopy or ultrasonography when using a percutaneous method [4]. Additionally, percutaneous suprapubic catheters are often limited by a smaller caliber (8–14 Fr) that is prone to occlusion. This is especially true in patients with 8–12 Fr biliary pigtail percutaneous catheter inserted by Interventional Radiology. Surprisingly, the use of a biliary catheter in the urinary tract has never been studied or FDA approved. The open abdominal approach under general anesthesia is still preferred by some urologists as it allows placement of a large caliber (20–24 Fr) catheter and avoids bowel injury [10]. The disadvantages to open abdominal approach are the need for abdominal dissection, length of the procedure, postoperative pain and convalescence that may necessitate hospital admission, placing a health and financial burden on the patient and healthcare system. Hence, there is a need for a medical device that allows a safe, efficient, and accurate method of suprapubic catheter placement particularly in the obese patient that comprised 45.6 % of the population in our study and 35 % of the US population [11].

Prior abdominal surgery without simultaneous imaging (ultrasound, fluoroscopy, CT scan or even laparoscopy) is considered by some to be a contraindication to percutaneous SPC placement due to surgical adhesions that can lead to bowel injury. In our study, the T-SPeC^®^ was placed safely without any intra-operative imaging. Preoperative imaging was needed in less than 10 % of patients. We did not experience a single bowel injury despite treating a population with previous gastrointestinal surgery (41.2 %), urological surgery (53.5 %) and gynecological surgery (71.9 %). Previous abdominal surgery did not put the patient at risk of bowel injury in our study as evident by the absence of bowel perforation in this study. However, we were selective in whom we would offer SPC placement using the T-SPeC^®^ device and triaged 12 high-risk patients during our study period to an open abdominal SPC placement. We believe the unique design of the T-SPeC^®^ device allows safe suprapubic catheter placement in many patients with abdominal adhesions, but this currently is listed by the manufacturer as a contraindication for T-SPeC^®^ insertion.

We found the T-SPeC^®^ Surgical System to be safe and effective even in morbidly obese patients. Although the percutaneous trocar in the ‘outside-in’ kit may not always reach the bladder in the obese population, we have not found a single patient that exceeded the limits of the T14 device despite patients with BMI as great as 50 kg/m^2^ [12]. Our T-SPeC^®^ intra-operative (1.8 %) and 30-day postoperative (10.5 %) complication rate compares favorably with the percutaneous SPC complication rate noted in the Ahluwalia study, 10 and 19 %, respectively [4]. Moreover, unlike Ahluwalia et al. [4], we did not have any bowel injuries or deaths. While not only safe, the T-SPeC^®^ procedure takes average of only 3.6 min which compares favorably to an open abdominal approach. The primary disadvantage of the T-SPeC^®^ device is that a patent urethra is required. Therefore, five patients were triaged to a percutaneous approach during the study period. Also the patient needs to be positioned in the lithotomy position that can be challenging in patients with lower extremity contractures.

The primary study limitation was that 88 % of patients had concomitant pelvic surgery. We believe the feasibility; benefits and postoperative complications would have been better demonstrated if the procedure was done in isolation. Other study limitations include a single surgeon, single institution experience and the lack of a control group. Consequently, we propose a multicenter study to demonstrate the outcome in isolated procedures. If these results prove encouraging, then a head-to-head, randomized, multicenter trial comparing operative time, success, safety, reliability and cost of T-SPeC^®^ versus percutaneous SPC would be valuable.

## Conclusion

The T-SPeC^®^ device is a disposable medical device that allows a novel, simple and accurate method of placing a SPC from an ‘inside-to-out’ approach in a heterogeneous group of patients. Our US series of 114 patients is the largest series to date and demonstrates that the T-SPeC^®^ device could be placed safely and efficiently in a large group of patients requiring a suprapubic catheter. We believe that future studies will further demonstrate that the T-SPeC^®^ has inherent advantages over current methods of SPC placement.
